# A multi-dimensional approach combining HPLC, ion mobility and high resolution mass spectrometry for investigating transition metal complexes

**DOI:** 10.1038/s41598-026-57597-w

**Published:** 2026-06-16

**Authors:** Laura Zellner, Julian Süß, Samuel Vorbach, Uwe Monkowius, Markus Himmelsbach, Stephan Hann, Christian W. Klampfl

**Affiliations:** 1 Institute of Analytical and General Chemistry, Johannes Kepler University Linz, Altenberger Strasse 69, 4040 Linz, Austria; 2 School of Education, Chemistry, Johannes Kepler University Linz, Altenberger Strasse 69, 4040 Linz, Austria; 3 Institute of Catalysis, Johannes Kepler University Linz, Altenberger Strasse 69, 4040 Linz, Austria; 4 Institute of Inorganic Chemistry, Johannes Kepler University Linz, Altenberger Strasse 69, 4040 Linz, Austria; 5 Department of Natural Sciences and Sustainable Resources, Institute of Analytical Chemistry, University of Natural Resources and Life Sciences, Muthgasse 18, 1190 Vienna, Austria

**Keywords:** Drift-tube ion-mobility mass spectrometry, HPLC, Transition metal complexes, Organometallics, Collision cross sections, Chemical biology, Chemistry

## Abstract

Organometallic complexes play a central role in catalysis and medicinal chemistry, yet their analytical characterization via mass spectrometry remains challenging due to their reactivity, susceptibility to fragmentation and the resulting overlapping isotopologue patterns, especially in complex reaction mixtures. Herein, we present a high-performance liquid chromatography ion mobility mass spectrometry workflow for the separation and analysis of group-six metal complexes. Chromatographic separation was combined with variation of electrospray ionization source conditions, allowing fragmentation behavior to be tuned according to specific analytical requirements. Ion mobility spectrometry provided an additional separation dimension that enabled the determination of collision cross sections as molecular descriptors for all investigated complexes. Analysis of the ion mobility data revealed systematic, metal-dependent trends consistent with expected structural differences. These correlations facilitated the assignment of the central metal even for species of low abundance or those lacking characteristic isotopologue patterns. Overall, this work demonstrates that the combination of chromatographic separation and ion mobility-based trend analysis provides a versatile platform for the characterization of organometallic species.

**Supplementary Information:**

The online version contains supplementary material available at https://doi.org/10.1038/s41598-026-57597-w.

## Introduction

Organometallic compounds are defined as substances that contain at least one direct metal-carbon bond. Its nature depends on the metal, its oxidation state and the organic moiety yielding compounds with M–C bonds ranging from highly polar and reactive to practically nonpolar and very inert. Additionally, further ligands can be introduced bonded via heteroatoms featuring “classical” coordination covalent bonds. This makes this class of compounds highly versatile with applications in all areas of chemistry. For example, organometallic compounds play an important role in industry as catalysts, in optoelectronic devices, as pharmaceutical active agents as well as potential sensor materials for biological applications and are therefore widely researched nowadays^1–4^. For catalytic reactions, maybe the most widespread and economically most significant application of organometallic compounds, mainly transition-metal complexes like Pd, Co, Cr or Rh complexes are used on ton scale reactions, e.g. for oxidations, carbonylation of methanol and hydrogenation reactions^1^. In chemotherapy, a variety of metal complexes, amongst others, based on Pt, Ru, Ir, Rh and Re are readily applied or researched^5,6^. In addition to catalytic and antitumor properties, some organometallics show antibacterial^7,8^ or antifungal properties^9^ while some archaea even utilize Mo and W for enzymatic processes^10^.

Mass spectrometry (MS) has already been established in the characterization and identification of organometallic molecules and is also applied to study reaction mechanisms and dynamics^11–14^. Recent examples are the speciation of gold(I) fluorinated benzenethiolates using electrospray ionization MS (ESI-MS) in combination with inductively coupled plasma atomic emission spectroscopy^15^ or mechanistic insights into the catalytic oxidative coupling of aryl lithium compounds^16^. Especially ESI-MS is applied preferentially, as it is a soft ionization technique that produces less fragmentation than harder techniques such as electron ionization, making it well suited for thermally labile species. Additionally, in MS many metal complexes can easily be recognized as the characteristic isotopologue patterns of many metals significantly facilitate their identification^13,17,18^.

Despite the soft ESI ionization, fragmentation is still frequently observed for metal complexes due to their labile character^19,20^. These additional signals with usually broad isotopologue footprints complicate the spectral interpretation. Besides these in-source fragments, often remainders of synthesis starting materials or byproducts additionally crowd the mass spectra as organometallic compounds are often highly reactive and prone to oxidation or thermal, photochemical and hydrolytic degradation, as for example seen for a tungsten complex previously synthesized by our group, which in solution rapidly oxidized^21^. To elucidate the origin of the signals, whether they stem from fragmentation or distinct species, high-performance liquid chromatography (HPLC) is the method of choice.

The use of HPLC for separation of organometallic compounds has been established some time ago^22,23^. This analytical method seems ideal for many metal complexes as the separation is performed at ambient temperature, rapidly, in the dark and with a degassed mobile phase that can be chosen based on the nature of the complex.

To further enhance selectivity and support the assignment of compounds detected by HPLC-MS, ion mobility spectrometry (IMS) can be employed as an additional separation dimension. In IMS, ions are separated in the gas phase according to their mobility through a neutral buffer gas under the influence of an electric field^24,25^. The mobility depends on the ion’s size, shape and charge and thus connects structural to mass-to-charge information. The resulting drift times can be converted to collision cross sections (CCS), which are size parameters related to the averaged momentum transfer impact area of the molecule, to yield instrument-independent structural descriptors^25^. This added dimension is useful for identifying conformational trends across different organometallic complexes and for relating fragmentation products to their corresponding parent species.

The applicability of IMS to organometallic compounds has already been demonstrated in a number of studies^26–33^. For example, IMS has been used to characterize the structures and gas-phase configurations of organoruthenium anticancer complexes^30,31,33^, to distinguish isomeric metal complexes were DOSY NMR was less effective^29^ and to detect catalytic intermediates in dual gold catalysis^27^. Combined computational and IMS approaches have also provided mechanistic insight into cationic ruthenium-catalyzed hydroboration reactions^28^ and the structural complexity of metal complexes formed with glyphosate derivatives^32^. These studies highlight how IMS can be used to separate isomeric complexes, probe ligand environments and gain insight into the structural complexity and reaction pathways of metal-ligand systems.

Beyond structural investigation of individual species, IMS also has the potential to show systematic relationships in mobility of similar complexes, which for instance contain the same metal or ligands^26^. A convenient framework for describing such systematic trends is the representation of ions in conformational space, commonly visualized by plotting CCS as a function of mass to charge (*m/z*) ratio^34^. In this representation, ions with similar overall architectures occupy characteristic regions, reflecting fundamental relationships between mass, size and shape. This concept has been extensively applied in the field of biomolecular MS, where different molecular classes, such as peptides, proteins, carbohydrates and nucleic acids are known to populate distinct regions in the CCS-*m/z* space depending on their gas-phase packing efficiency^34–36^. While the molecular composition and bonding of organometallic complexes differ fundamentally from biomolecules, their 3D architecture similarly influences gas-phase mobility, suggesting that CCS trends can be analyzed to reveal systematic structural relationships within families of metal complexes.

In the present study, we report a tailored HPLC-IM-MS workflow for a set of 17 closely related group six transition metal complexes sharing a common carbonyl-based ligand framework. Source conditions were systematically explored to demonstrate that fragmentation behavior can be tuned to suit specific analytical needs. In addition, ion mobility data were used to examine conformational space and to assess systematic trends in CCS across related complexes. Such trends can be used to support the classification of unknown or low-abundance species based on their mobility, while the multidimensional separation also facilitates the detection of reaction byproducts. This integrated approach combines multidimensional separation, controlled fragmentation and structural trend analysis, providing a versatile platform for the characterization of organometallic species.

## Materials and methods

### Chemicals and materials

Acetonitrile, methanol, acetone and ammonium formate (≥ 99%, HPLC grade) were provided by VWR (Vienna, Austria). Formic acid (HPLC grade) was acquired from Merck (Darmstadt, Germany). Ultrapure water was obtained from a Milli-Q water purification system (Millipore, Bedford, MA, USA).

### Complex synthesis

A total of 17 group six transition-metal complexes were synthesized and analyzed. The complexes all share a hexacarbonyl complex as precursor and are coordinated to various nitrogen and phosphorous containing ligands. The investigated complexes are described by their molecular formulas, exact masses and structures as listed and shown in Table S1 and Figure S1 (exact masses were calculated with *enviPat*^37^. All metal complexes were synthesized according to previously reported procedures^21,38–42^.

In addition to the clearly defined complexes mentioned above, crude reaction mixtures were analyzed. The synthetic procedure for these complexes was based on published procedures^38,43,44^. The reactions underwent minimal work-up.

### HPLC-IM-QTOF-MS

The synthesis products were dissolved in acetone, methanol or in tetrahydrofuran, depending on their solubility, and filtered (0.45 μm pore size) prior analysis. The separation of the samples was achieved by means of HPLC (Agilent 1260, Agilent Technologies, Waldbronn, Germany) equipped with a Phenomenex Luna C18 (4.6 × 150 mm, 3 μm particle size) column. The mobile phase comprised water (solvent A) and methanol (solvent B). The gradient elution program commenced at 70% solvent A and 30% solvent B, with a subsequent linear increase to 80% solvent B within 1.5 min. These conditions were held until 4 min. Over the next 6 min solvent B was linearly increased to 100%. Next, a cleaning step with 100% solvent B was employed before at 13.1 min the solvent ratios were returned to their initial conditions, finishing the run with a total time of 15 min. The flow rate was maintained at 0.5 mL min^− 1^, the column oven was heated to 30 °C and the injection volume was set to 10 µL. Water: methanol (1:1%(v/v)) with 1% formic acid and 100 mM ammonium formate was infused post-column using an isocratic pump with a flow rate of 0.05 mL min^− 1^ to enhance ESI ionization.

The HPLC system was coupled to a high-resolution drift tube IM-QTOF-MS (Agilent 6560 A, Agilent Technologies, Waldbronn, Germany) equipped with an Agilent Dual Jet Stream Electrospray Ionization source, which was operated in positive ion mode. The drying gas temperature and sheath gas temperature were both set at 275 °C, with flow rates fixed at 11 L min^− 1^ (both nitrogen). The nebulizer pressure was kept at 40 psi, the capillary and nozzle voltages were set at 1500 V and 1000 V respectively, while the fragmentor voltage was maintained at 250 V.

The IM-QTOF-MS was tuned in the fragile ion mode and operated with 5-bit multiplexing. The trap fill time was set to 1800 µs, trap release time to 250 µs, frame rate was 1.0 frames sec^− 1^, transient rate was 17 transients frame^− 1^ and maximum drift time was set to 60 ms. The advanced parameters comprised the following settings: drift tube entrance voltage of 1567 V, drift tube exit voltage of 217 V, rear funnel entrance of 210.5 V and rear funnel exit of 38 V ^45^. All IM measurements were performed under fixed ion source conditions.

The influence of ion source parameters was systematically evaluated by varying each parameter over representative low, medium and high settings resulting in a total of 21 experimental conditions. Therefore, the complexes were measured using flow injections with 80% methanol and 20% water as mobile phase at a flow rate of 0.3 mL min^− 1^. The screening focused on capillary voltage, fragmentor voltage, nozzle voltage, nebulizer pressure, drying gas temperature and sheath gas temperature.

The peak areas of the complexes were recorded and the effect of each source parameter was calculated relative to the medium setting (defined as 100%). Average peak areas obtained at low and high settings were expressed as percentage changes compared to the medium setting. Additionally, selectivity for ionizing intact complexes versus in-source fragmentation was assessed.

### Data processing

Data evaluation/processing was done using Agilent MassHunter Qualitative Analysis B.07.00, PNNL PreProcessor 5.0 (2024.05.31) and IM-MS Browser 10.0.1.

The PNNL PreProcessor software was used for demultiplexing the IM data files. The data were calibrated with the recorded single field tune using IM-MS browser. The drift times and the ^DT^CCS_N2_ were determined by performing a feature extraction for single field calibration data in the IM-MS browser using the monoisotopic masses of the metal complexes. Complexes that were not found by automated feature extraction were determined manually. In all cases, measured arrival times were transformed into ^DT^CCS_N2_ values according to a single field calibration approach, as stated by Stow et al. ^45^, following Eq. (1):

1 $$\:{t}_{A}=\frac{\beta\:}{z}\sqrt{\frac{{m}_{i}}{{m}_{i}+{m}_{B}}}{}^{\mathrm{D}\mathrm{T}}{\mathrm{C}\mathrm{C}\mathrm{S}}_{{\mathrm{N}}_{2}}+{t}_{\mathrm{f}\mathrm{i}\mathrm{x}}$$

with $\:{t}_{A}$ arrival time, $\:\beta\:$ slope of the single field calibration, * z* ion charge, $\:{m}_{i}$ ion mass, $\:{m}_{B}\:$neutral gas mass and $\:{t}_{fix}$ intercept of the single field calibration.

For the construction of the conformational landscape only ^DT^CCS_N2_ values of singly charged [M]^+^ or [M + H]^+^ cations were considered. In addition to the investigated complexes, all detected in-source fragments and degradation products were included. ^DT^CCS_N2_ were measured five times and the mean value was used for plotting.

## Results and discussion

### Chromatographic separation

A chromatographic method was developed that allows to separate the products from 17 transition metal complex syntheses, which are listed in Table 1. One representative complex (complex **W-5**) that shares the common carbonyl-based metal precursor of all investigated species, was selected as a model compound. Based on this complex, the stationary phase, mobile phase composition, eluent additives, column temperature and the mobile phase gradient were optimized. Among the tested columns, the Phenomenex Luna C18 provided the best peak shapes and resolution. In the course of the optimization, both methanol and acetonitrile were tested as eluents. However, due to the coordinative properties of acetonitrile, which result in dynamic ligand exchange for some of the complexes, methanol was selected for the final method. To minimize interactions between additives and the metal complexes, formic acid and ammonium formate were infused post-column for enhancing ESI ionization. Furthermore, the experiments revealed that increasing the temperature of the column oven enhanced peak symmetry. However, this was accompanied by an increase in ligand dissociation or oxidation of the analytes. Consequently, the final temperature was maintained at 30 °C. Given the predominantly nonpolar nature of the metal complexes, a gradient which rapidly increased the content of organic eluent provided the best retention and resolution.

**Table 1:** Chromatographic figures of merit for the detected W, Mo and Cr complexes.

Abbreviation	Complex	RT / min	Peak width / min	Symmetry / -	FWHM / min
**W-1**	CpW(CO)_2_PN	5.4	0.6	2.4	0.1
**W-2**	CpW(CO)_2_PP	5.6	0.6	2.8	0.1
**W-3**	CpW(CO)_2_PPP*	5.9	0.5	2.9	0.1
**W-4**	W(ACN)_3_(CO)_3_	6.2	0.5	1.2	0.1
**W-5**	W(Py)_2_(CO)_4_	6.9	0.6	1.8	0.1
**W-6**	CpW(CO)_2_PPP	7.5	0.8	2.2	0.3
**Mo-1**	CpMo(CO)_2_PPP	7.5	1.3	3.0	0.3
**Mo-2**	Mo(Py)_2_(CO)_4_	8.1	0.6	1.9	0.2
**W-7**	W(CO)_4_PN	8.4	0.5	1.8	0.1
**Cr-1**	Cr(CO)_4_PN	8.7	0.8	1.0	0.2
**W-8**	W(iPrBiOx)(CO)_4_	9.3	0.6	2.0	0.1
**Mo-3**	Mo(iPrBiOx)(CO)_4_	10.0	0.4	1.6	0.1
**W-9**	IndPhosW	12.5	0.7	2.1	0.2
**Mo-4**	IndPhosMo	12.6	0.6	2.0	0.1
**Cr-2**	Cr(dppm)(CO)_4_	12.9	0.6	1.9	0.1
**W-10**	W(dppm)(CO)_4_	13.0	0.6	2.3	0.1
**Mo-5**	Mo(dppm)(CO)_4_	13.4	0.6	2.7	0.1

*RT* retention time, *FWHM* full width at half maximum

The final chromatograms exhibited distinct, well-defined and narrow peaks, demonstrating efficient separation of the complex species (Table 1, Supplementary Fig. S2). The improved resolution enabled the detection of synthesis byproducts, like a mono-pyridine species in the case of **W-5**, that were not observed by conventional analysis techniques, such as flow injection mass spectrometry.

**Fig. 1:**
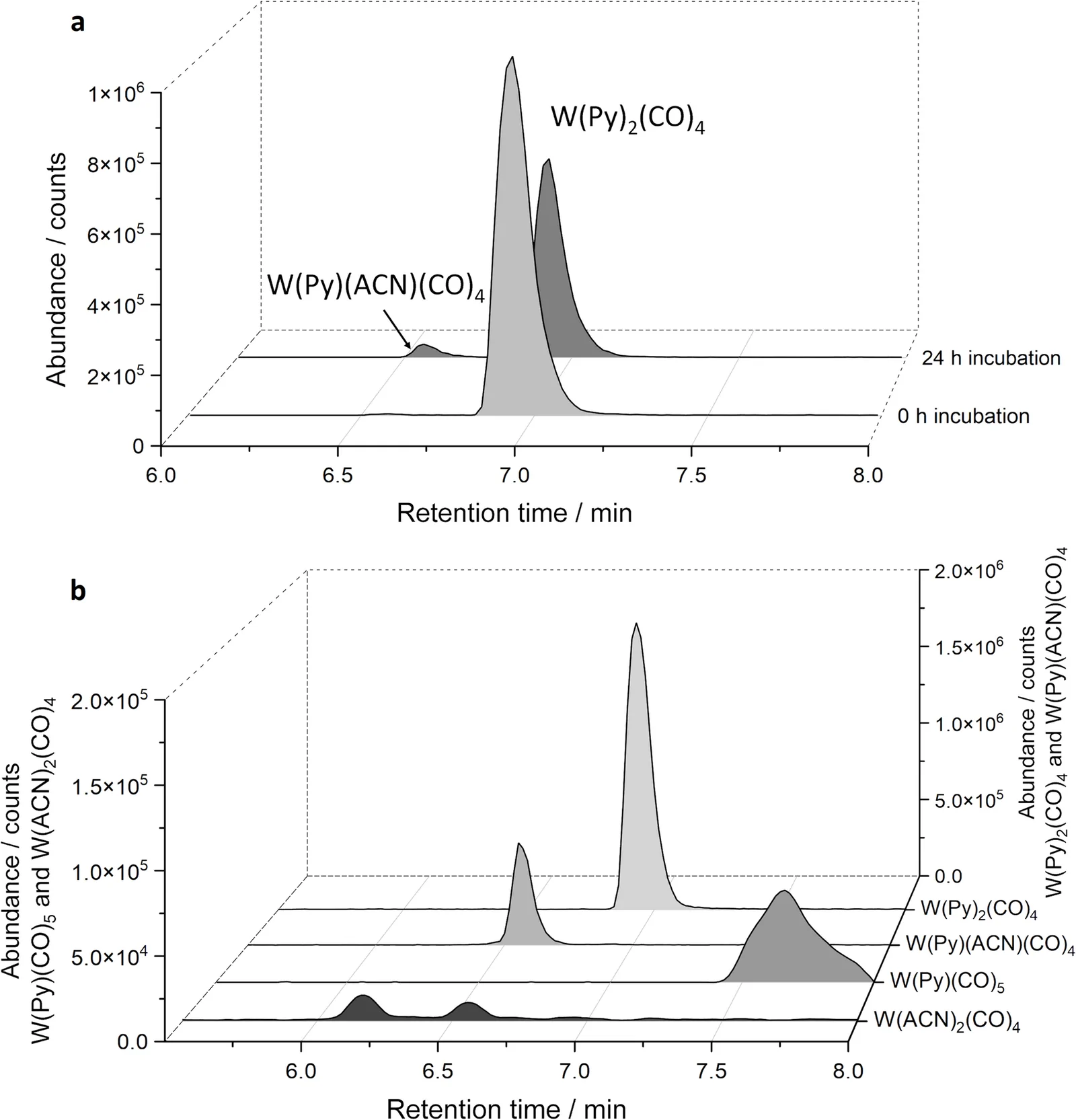
Extracted ion chromatograms of complex **W-5**. (**a**) Observed ligand exchange with acetonitrile for **W-5** (W(Py)_2_(CO)_4_) over time. (**b**) Extracted ion chromatograms of **W-5** and its byproducts, showing the resolving power of the method.

Also, on the example of **W-5**, the resolving power of the method can be demonstrated. Signals for mono- and di-pyridine species were clearly resolved, with considerably lower polarity for the singly substituted complex. When the reaction products were dissolved in acetonitrile, additional signals appeared, originating from ligand exchange between pyridine and acetonitrile. Over time, the intensity of the acetonitrile-substituted complexes increased, while the original pyridine-coordinated peaks gradually decreased (Fig. 1a). Consequently, the synthesized complexes were dissolved in acetone to rule out ligand exchange processes with acetonitrile. Nonetheless, despite the presence of two additional complex species, the method permitted full separation of all complexes (Fig. 1b).

### Mass spectrometry experiments

Following the optimization of the chromatographic separation, ESI-MS spectra of the herein investigated complexes were recorded on an IM-QTOF-MS. Most of the species were detected as single-charged cations without adduct formation or as protonated species (Supplementary Table S2). However, in the case of **W-3**,** W-4**,** Mo-2** and **Mo-3**, the ionization took place in the course of the dissociation of a carbonyl or acetonitrile ligand. For complex **Mo-4**, intact ions were observed only as ammonium adducts, whereas protonated ions were associated with carbonyl dissociation. Simulated isotope pattern for all of the complexes agreed well with the experimental ones, es exemplarily seen for **Cr-2**, **Mo-5** and **W-10** in Figures S3-S5, which confirms the elemental composition of the analytes.

**Fig. 2:**
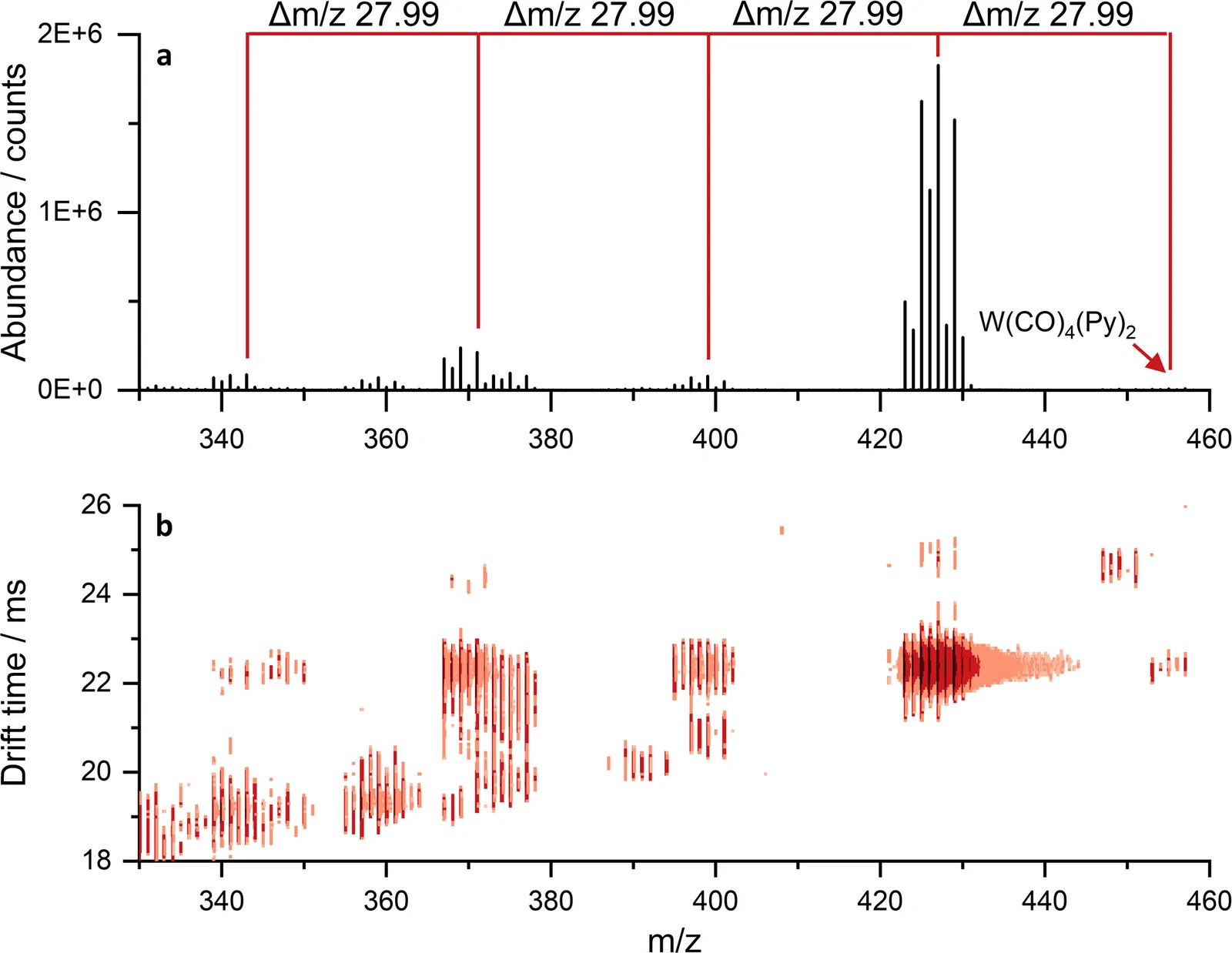
Mass and drift spectrum showing the in- and post-source fragmentation of **W-5**. (**a**) Mass spectrum of **W-5** (W(Py)_2_(CO)_4_). Several signal can be seen that have a mass difference of *m/z* 27.99, corresponding to the loss of a carbonyl ligand during MS measurement. (**b**) Drift time vs. *m/z* spectrum of **W-5** (W(Py)_2_(CO)_4_), revealing post drift tube fragmentation as the fragments share the drift time with the molecular peak at *m/z* 455.02.

Furthermore, the mass spectra contained various additional signals exhibiting the characteristic isotopologue pattern of the metal. The *m/z* of these signals differed from that of the intact complex by the neutral mass of one of the ligands, most often a carbonyl group (Fig. 2a). Since the retention times of these signals coincided with those of the intact complex, it can be concluded that they are not distinct species, but rather, that the signals stem from in- or post-source fragmentation, which are frequently observed for labile compounds^20^.

To determine whether the observed fragmentation occurs in the ESI source or at a later stage, drift spectra can be examined. In the IM-QTOF-MS setup, the drift tube is located directly after the ESI source and before the quadrupole. Therefore, complexes that fragment in the source enter the drift tube as fragments and exhibit distinct drift times shorter than those of the intact complex. In contrast, if fragmentation occurs after the drift tube, the complexes pass through the tube intact and are assigned a single drift time. Subsequent fragmentation leads to signals with different *m/z* but the same drift time as the parent species. These signals appear as horizontal lines in the drift spectrum (Fig. 2b). A possible reason for the fragmentation post-source is the pressure gradient after the drift tube which might lead to dissociation^46^.

**Fig. 3:**
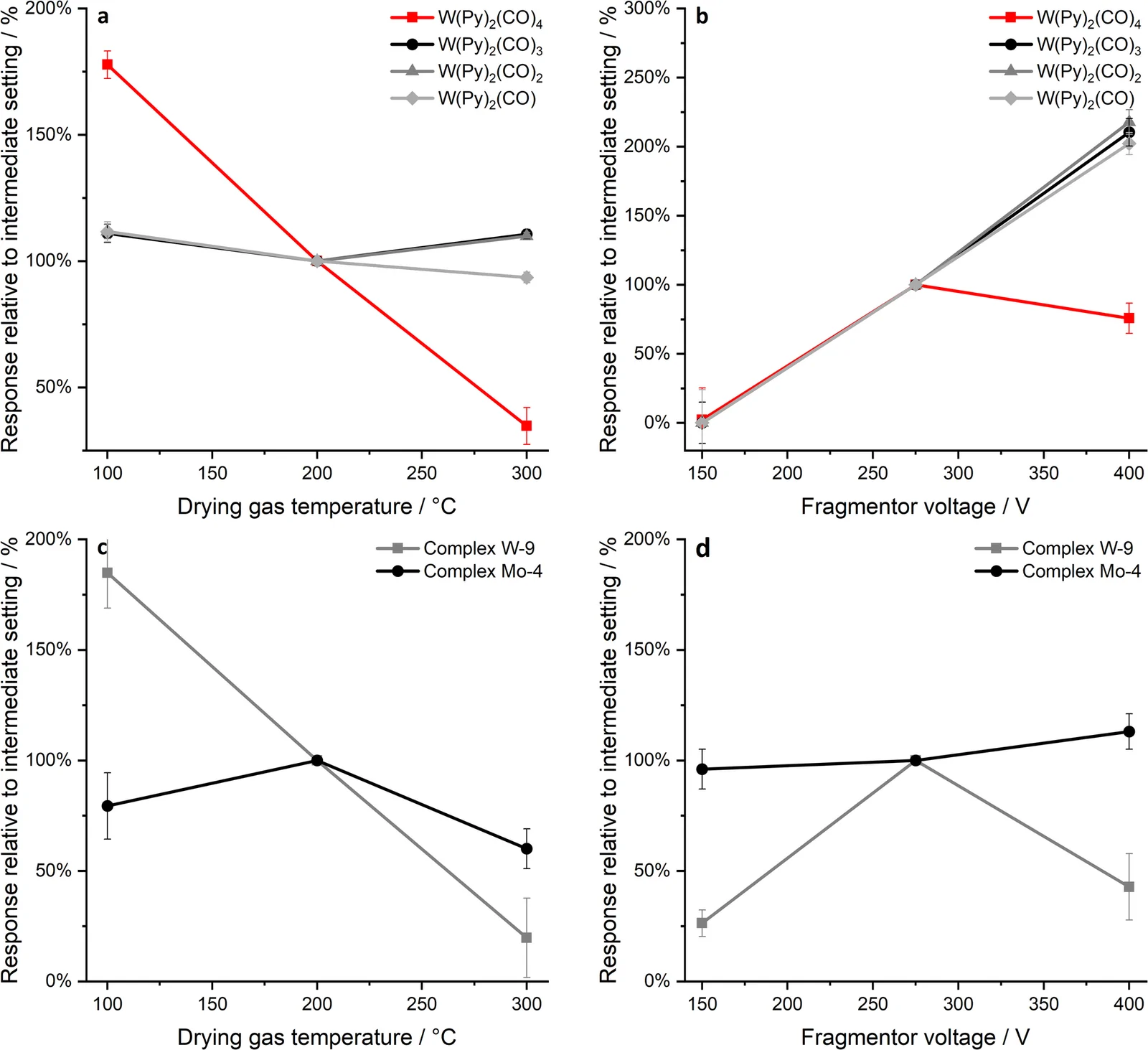
Source parameter experiments. Relative percentage change of the response upon variation of fragmentor voltage and drying gas temperature. The response obtained at intermediate source settings was defined as 100% and the relative percentage changes observed at low and high settings are shown. (**a**,**b**) **W-5** and its in-source fragments; (**c**,**d**) **W-9** and its Mo counterpart (**Mo-4**).

Since ESI ionization strongly influenced the obtained complex signals, the impact of the source parameters was screened. Figure 3 shows the percentual change of using a high or low setting relative to an intermediate setting for selected source parameters, with the red line representing the intact model complex **W-5**. The signal intensity of **W-5** was most influenced by drying gas temperature (Fig. 3a) followed by capillary voltage. Increasing either parameter resulted in pronounced signal intensity decreases. For the fragmentor voltage (Fig. 3b) and the nozzle voltage intermediate parameter settings yielded the highest response while the signal decreased when the voltages were set at too high or too low values. In contrast, low sheath gas temperature gave the best results. The effect of different nebulizer pressure settings was non-significant.

Next, the effect of the source parameters on in-source fragmentation was assessed by comparing the signal of **W-5** to the responses of its fragments lacking one, two or three carbonyl groups. Figure 3a shows that increasing drying gas temperature selectively decreased the response of the intact complex, which was also observed for capillary voltage. Furthermore, higher fragmentor voltages promoted ligand loss (Fig. 3b), while intermediate nozzle voltages favored the intact species.

Taking the other complexes into account, robust settings that benefit the ionization of all species were sought. A high fragmentor voltage consistently led to stronger signals for most complexes. In general, moderate to low nozzle voltage and nebulizer pressure improved responses. However, no universal setting could be identified for the drying gas temperature, sheath gas temperature or capillary voltage, as individual complexes exhibited markedly different responses to varying these parameters. Consequently, while a set of generally robust conditions can be defined, achieving maximum signal intensity requires fine-tuning of drying gas temperature, sheath gas temperature and capillary voltage for each complex individually.

Finally, the response of similar complexes that differ only in their core metal can be investigated with respect to varying source parameters. When comparing **W-9** with its Mo analogue (**Mo-4**) the former one was more sensitive to changes of the source parameter settings. While the Mo-complex remained mainly unaffected by varying the source settings, the W-complex exhibited significant differences in response to changes in capillary voltage, fragmentor voltage, nozzle voltage, nebulizer pressure, drying gas temperature and sheath gas temperature (Fig. 3c and d). This indicates that W complexes may require more careful source optimization to achieve maximized signal intensities. Furthermore, these findings again emphasize that, for best performance, source parameters should be optimized on a per-complex basis.

Overall, the screening experiments identified fragmentor voltage and drying gas temperature as the most influential parameters for maximizing signal intensity and preserving intact complexes. Consequently, a low drying gas temperature (~ 100 °C), moderately high fragmentor voltages, nozzle voltages and nebulizer pressures (275 V, 1000 V and 40 psi, respectively) and a low capillary voltage (1500 V) were selected as suitable conditions. Similar observations were reported by Chagunda et al. ^20^, who stated that low capillary voltages stabilize the complex signals, whereas higher source temperatures lead to reduced response^47^.

However, since our method’s chromatographic resolution allows us to distinguish in-source fragments from distinct chemical species, we increased the drying gas temperature in our experiments to slightly promote ligand loss and collect more ion mobility data. Additionally, a drying gas temperature of only 100 °C could cause desolvation issues during the more aqueous phase of the chromatographic gradient.

As our goal in this work was to establish a universally applicable method, we selected the following parameters for all subsequent experiments: 1500 V capillary voltage, 1000 V nozzle voltage, 250 V fragmentor voltage, 40 psi nebulizer pressure and 275 °C drying gas temperature and sheath gas temperature. With these settings all complexes showed good response with moderate fragmentation. Our findings highlight that the source parameters can be tuned to be fit-for-purpose. Slight adjustments to certain parameters allow to shift the focus from efficient ionization of the intact complex to promoting in-source fragmentation.

### Collision cross sections

In addition to that, analysis of the drift tube ion mobility (DTIM) data provided valuable structural insights by determining the ^DT^CCS_N2_ values of the separated species. Supplementary Table S2 shows the ^DT^CCS_N2_ values of all measured species. The sizes strongly correlate with the molar mass of the ligands. This trend is expected due to the increased steric bulk and mass associated with the addition of more or bigger ligands, which leads to larger molecular structures and hence higher ^DT^CCS_N2_ values.

**Fig. 4:**
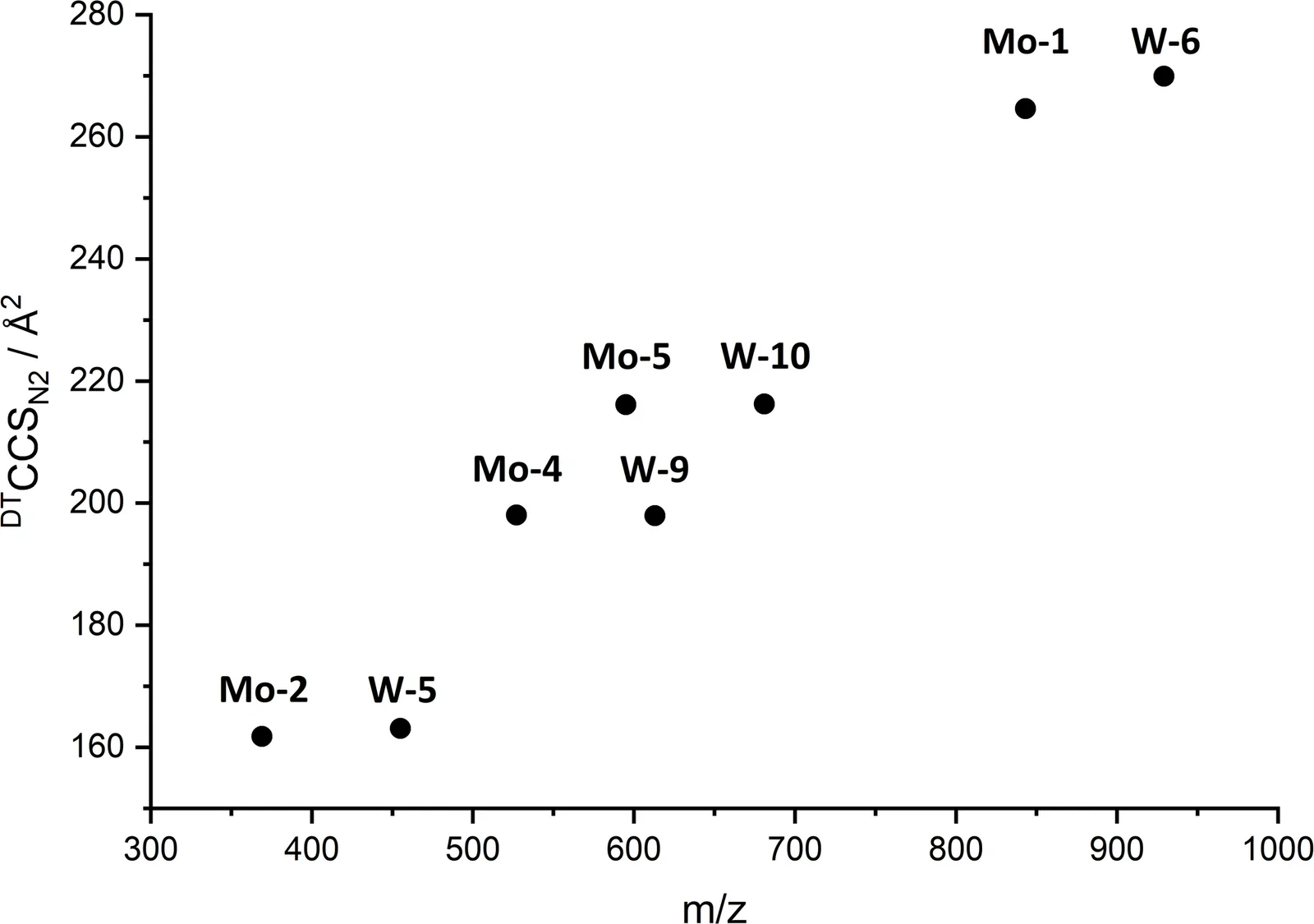
Comparison of ^DT^CCS_N2_ of Mo and W complexes. Complexes with the same ligands but different metal show almost similar ^DT^CCS_N2_ despite of a *m/z* difference of 86.05.

In contrast to expectations, Mo complexes do not exhibit smaller CCS compared to those bearing the larger W as metal center which can be seen when comparing **W-5** and **Mo-2**, **W-6** and **Mo-1** as well as **W-9** and **Mo-4** or **W-10** and **Mo-5** (Fig. 4). Interestingly, although the complexes bearing the same ligand but different metal vary by a *m/z* of 87.04, the ^DT^CCS_N2_ are very similar with differences < 2 Å^2^, highlighting the higher compactness of W complexes. This observation is consistent with expectations based on the similar atomic and ionic radii of W and Mo, which are influenced by the lanthanide contraction^10^. For complex pair **W-6** and **Mo-1**, the size difference is slightly more pronounced (roughly 5 Å^2^), which may be attributed to increased intramolecular flexibility arising from the dangling phosphine arm of the ligand (see Figure S1 for structures).

**Fig. 5:**
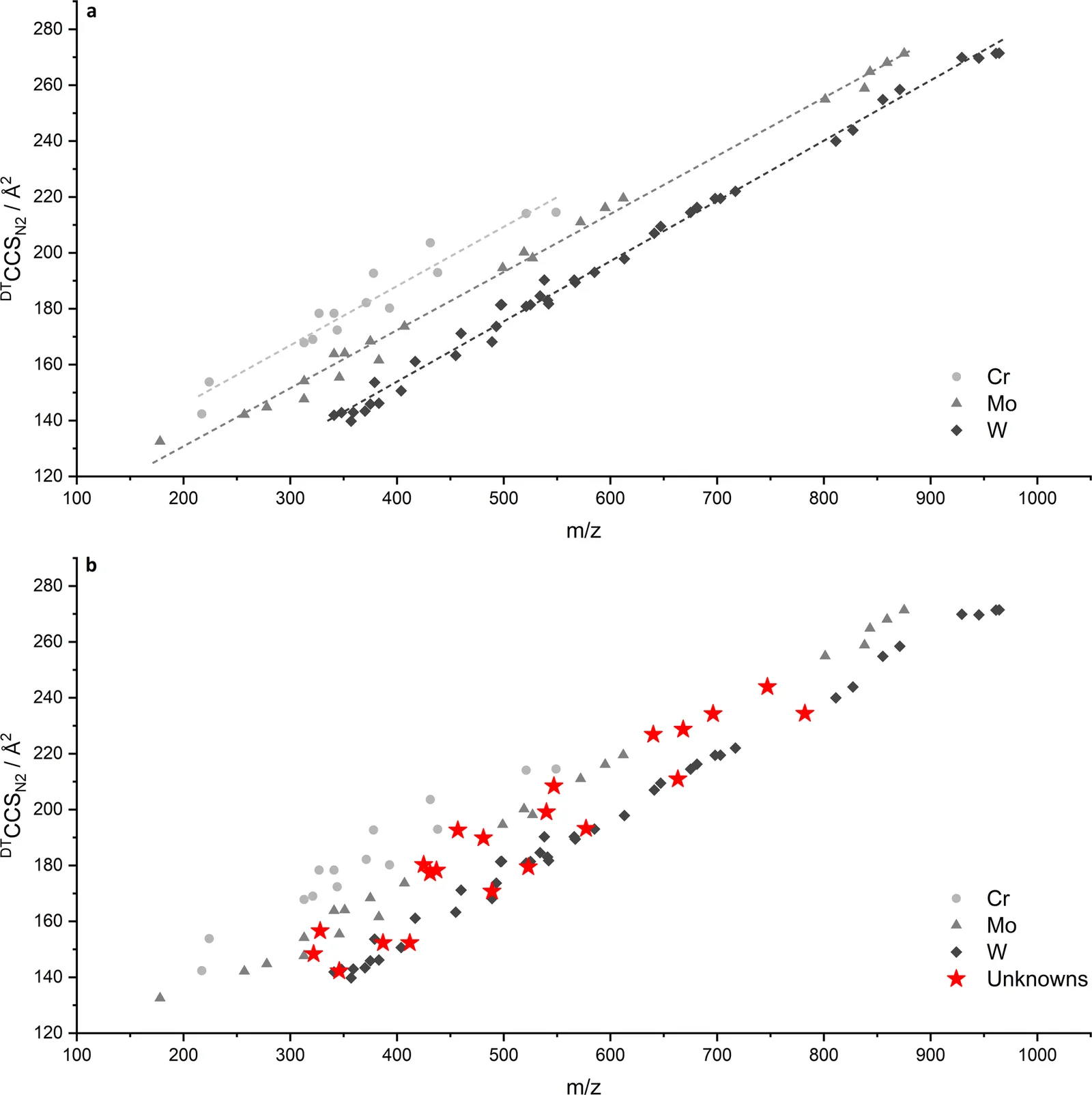
Conformational landscape of Cr, Mo and W complexes. (**a**) Plotting ^DT^CCS_N2_ vs. *m/z* for the investigated Cr, Mo and W species, linear trends related to the center metals are obtained. (**b**) Complexes with low intensity and hence unknown isotopic pattern or with unknown composition (Table S3) located in the conformational landscape of group six transition metals.

By plotting the *m/z* ratios of the complexes against their ^DT^CCS_N2_, a conformation landscape of the species can be obtained (Fig. 5a). In addition to the signals of the aforementioned complexes, this plot also includes all in-source fragments and byproducts that exhibited a metal-characteristic isotopologue pattern. As with biomolecules, where different biological classes are distinct in the landscape^34–36^, the group six transition metal complexes are distributed in space according to their metal center. At a given *m/z*, the relative ^DT^CCS_N2_ values increase in the order W < Mo < Cr, as complexes containing lighter metals must coordinate bulkier ligands to compensate for the lower metal mass, resulting in larger overall ^DT^CCS_N2_.

Examining the arrival times of the in-source fragmented complexes allows us to analyze the effect of losing individual ligands on the ^DT^CCS_N2_. The dissociation of a single carbonyl ligand is associated with a ^DT^CCS_N2_ decrease of around 5.4 ± 0.6 Å² and falls along the diagonal of the conformational landscape. This diagonal trend suggests that the complex conformation in the gas phase is not significantly affected when a carbonyl ligand is lost.

Finally, the metal ^DT^CCS_N2_ versus *m/z* trends aid in the investigation of byproducts with low signal intensity and a poorly recognizable isotopologue pattern. Because metal complex signals deviate from the primary trend of organic molecules (Supplementary Fig. S6), these features can be readily distinguished and investigated in relation to their respective metal centers. This approach was validated by analyzing several crude reaction mixtures stemming from attempts in synthesizing so far not known W or Mo complexes. These mixtures contained multiple unknown products or byproducts for which the sum formulas could not be deduced from the reaction pathway (Supplementary Table S3). In each case, the observed ^DT^CCS_N2_ values immediately indicated the identity of the metal center, when compared to our observed trends (Fig. 5b, Supplementary Table S3). For instance, faint W signals were detected in an Mo sample, likely due to trace W(CO)₆ contamination of the Mo(CO)₆ precursor. Although this byproduct’s isotopologue pattern (*m/z* 386.89) was inconclusive, its ^DT^CCS_N2_ value coincided with the W trend. Likewise, two other products were annotated in reaction mixtures 5 and 6 as well as a Mo contamination was found in reaction 6.

### Complex degradation products

Apart from complex signals stemming from in-source fragmentation, which share the same retention time as the intact complex, additional characteristic signals were observed at different retention times. In particular, for complexes **Mo-1** and **W-6**, that contain multiple phosphorous atoms, species with an *m/z* + 16 were frequently detected, most likely resulting from phosphorous oxidation. While these compounds are relatively stable under atmospheric conditions, they tend to oxidize quite rapidly when in solution as shown by Vielhaber et al. ^21^. According to the published structure of the W complex, the oxidation happens at the non-binding moieties of the ligand^21^. Moreover, signals corresponding to complexes containing a second additional oxygen were observed.

Supplementary Figure S7 shows the extracted ion chromatograms of the **Mo-1** and **W-6** species with one or two additional oxygen atoms. Oxidation of a single phosphorous atom increases the polarity of the complex leading to an earlier retention time. However, addition of a second oxygen does not significantly further increase polarity. As a result, the species containing two additional oxygen atoms largely coelute with the singly oxidized ones. IM measurements further supported the assignment of these degradation products, as they displayed reproducible ^DT^CCS_N2_ values distinct from those of the intact parent complexes. These species were included in the conformational landscape analysis (“Collision cross sections”) and were found to align with the same metal-dependent mobility trends observed for the intact complexes and in-source fragments.

## Conclusions

In this work, we developed a HPLC based separation method for group six transition metal complexes that allows subsequent characterization by IM-QTOF-MS. We showed that optimization of the ESI parameters can minimize in-source fragmentation and ensure reliable detection of intact complexes. Comprehensive ^DT^CCS_N2_ measurements revealed trends that correlate with the identity of the central metal and provided insight into complex geometries. As a result, the ^DT^CCS_N2_ values allow assignment of the metal center, even in reaction mixtures containing unknown or unexpected species with poorly resolved isotopologue patterns.

These findings highlight the utility of combining chromatographic separation with ion mobility and high-resolution MS for structural understanding of metal-ligand complexes. The clear ^DT^CCS_N2_-metal correlations provide a basis for a tentative assignment strategy that remains robust against fragmentation processes and capable of handling low-intensity signals.

## Supplementary Information

Below is the link to the electronic supplementary material.

## Data Availability

The datasets generated during the current study are available from the corresponding author on reasonable request.
