# Supplementary material for: A multi-dimensional approach combining HPLC, ion mobility and high resolution mass spectrometry for investigating transition metal complexes
Source: Sci Rep. 2026 Jun 16;16:27625. doi: 10.1038/s41598-026-57597-w (PMC13538485; doi:10.1038/s41598-026-57597-w)
Supplement: Supplementary file 1 [file 41598_2026_57597_MOESM1_ESM.pdf]

SUPPLEMENTARY INFORMATION

A MULTI-DIMENSIONAL APPROACH COMBINING HPLC, ION MOBILITY AND HIGH RESOLUTION MASS SPECTROMETRY FOR INVESTIGATING TRANSITION METAL COMPLEXES

Laura Zellner[1*], Julian Süß[2,3], Samuel Vorbach[3], Uwe Monkowius[2,4], Markus Himmelsbach[1], Stephan Hann[5], Christian Klampfl[1]

[1] Johannes Kepler University Linz, Institute of Analytical and General Chemistry, Altenberger Strasse 69, 4040 Linz, Austria

[2] Johannes Kepler University Linz, School of Education, Chemistry, Altenberger Strasse 69, 4040 Linz, Austria

[3] Johannes Kepler University Linz, Institute of Catalysis, Altenberger Strasse 69, 4040 Linz, Austria

[4] Johannes Kepler University Linz, Institute of Inorganic Chemistry, Altenberger Strasse 69, 4040 Linz, Austria

[5] BOKU University, Institute of Analytical Chemistry, Department of Natural Sciences and Sustainable Resources, Muthgasse 18, Vienna 1190, Austria

*Correspondence to Laura Zellner (E-mail: laura.zellner@jku.at), Tel +43 732 2468 9707

Table of Contents

S1 Complex characteristics 3
S1.1 Structure and composition 3
S1.2 Chromatographic separation 5
S1.3 Isotopic pattern 7
S2 Ion mobility measurements 8
S2.1 Trend of metal complexes in IM-MS 8
S2.2 IM-MS data of unknown complexes 9
S3 Complex degradation products 10
S4 References 10

Overview of Figures

Figure S1: Structures of the herein investigated complexes. 4
Figure S2: Extracted ion chromatograms of the herein investigated complexes. 5
Figure S3: Experimental and predicted isotopic pattern for **Cr-2**. 7
Figure S4: Experimental and predicted isotopic pattern for **Mo-5**. 7
Figure S5: Experimental and predicted isotopic pattern for **W-10**. 8
Figure S6: Drift time trends of organic molecules versus metal complexes. 8
Figure S7: Extracted ion chromatograms of oxidized byproducts.. 10

Overview of Tables

Table S1: Sum formulas and monoisotopic masses of the investigated metal complexes. 3
Table S2: Charged species, drift times and $^{DT}CCS_{N2}$ of the investigated complex species. 6
Table S3: Isotopologue pattern and assigned metal center for unknown complexes. 9

S1 Complex characteristics

S1.1 Structure and composition

Table S1: Sum formulas and monoisotopic masses (calculated using enviPat [1]) of the herein investigated group six transition metal complexes.

Abbreviation	Complex	Sum formula	Monoisotopic mass / Da
**W-1**	$CpW(CO)_2PN$	$C_{21}H_{21}NO_2PW$	534.0819
**W-2**	$CpW(CO)_2PP$	$C_{33}H_{29}O_2P_2W$	703.1152
**W-3**	$CpW(CO)_2PPP$*	$C_{41}H_{38}O_2P_3W$	839.1594
**W-4**	$W(ACN)_3(CO)_3$	$C_9H_9N_3O_3W$	391.0153
**W-5**	$W(Py)_2(CO)_4$	$C_{14}H_{10}N_2O_4W$	454.0150
**W-6**	$CpW(CO)_2PPP$	$C_{48}H_{44}O_2P_3W$	929.2063
**Mo-1**	$CpMo(CO)_2PPP$	$C_{48}H_{44}O_2P_3Mo$	843.1608
**Mo-2**	$Mo(Py)_2(CO)_4$	$C_{14}H_{10}N_2O_4Mo$	367.9695
**W-7**	$W(CO)_4PN$	$C_{18}H_{16}NO_4PW$	525.0326
**Cr-1**	$Cr(CO)_4PN$	$C_{18}H_{16}NO_4PCr$	393.0222
**W-8**	$W(iPrBiOx)(CO)_4$	$C_{16}H_{20}N_2O_6W$	520.0831
**Mo-3**	$Mo(iPrBiOx)(CO)_4$	$C_{16}H_{20}N_2O_6Mo$	434.0375
**W-9**	IndPhosW	$C_{28}H_{22}ClO_2PW$	640.0555
**Mo-4**	IndPhosMo	$C_{28}H_{22}ClO_2PMo$	554.0100
**Cr-2**	$Cr(dppm)(CO)_4$	$C_{29}H_{22}O_4P_2Cr$	548.0398
**W-10**	$W(dppm)(CO)_4$	$C_{29}H_{22}O_4P_2W$	680.0503
**Mo-5**	$Mo(dppm)(CO)_4$	$C_{29}H_{22}O_4P_2Mo$	594.0047

Cp = cyclopentadiene;
PP = 1,2-bis(diphenylphosphino)ethane;
ACN = acetonitrile;
iPrBiOx = isopropyl bisoxazoline;
dppm = bis(diphenylphosphino)methane
PN = 2-(diphenylphosphino)ethylamine;
PPP = 1,1,1-tris(diphenylphosphinomethyl)ethane;
Py = pyridine;
IndPhos = (diphenylphosphino)indene;

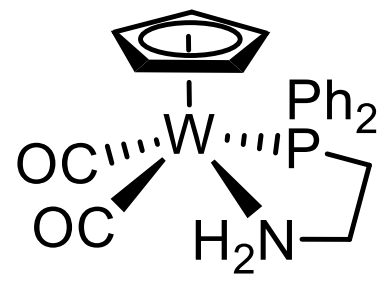

W-1

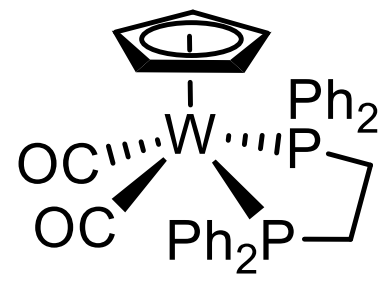

W-2

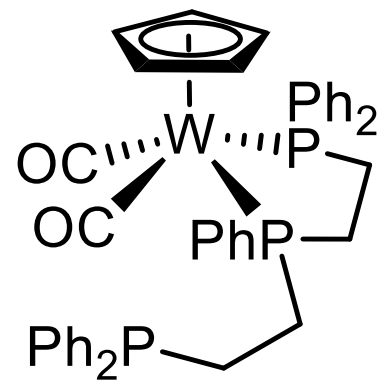

W-3

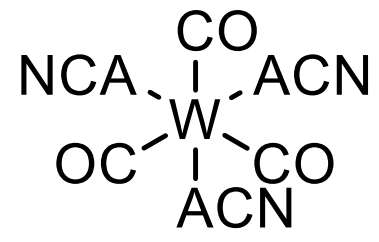

W-4

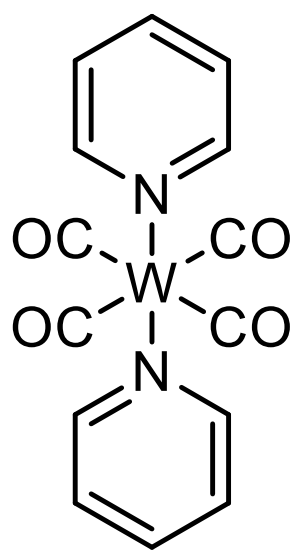

W-5

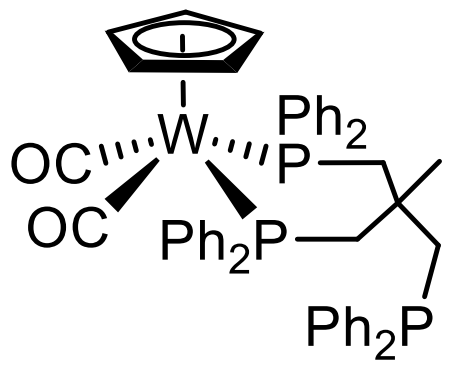

W-6

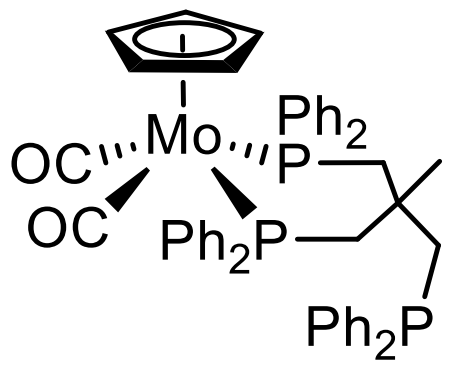

Mo-1

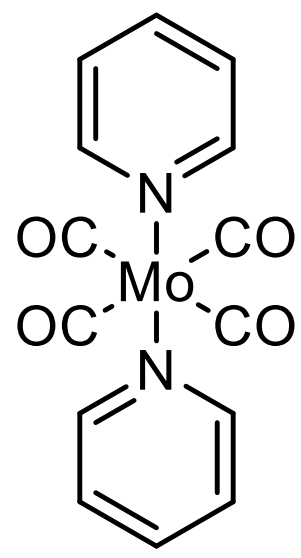

Mo-2

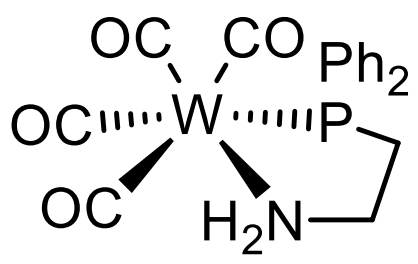

W-7

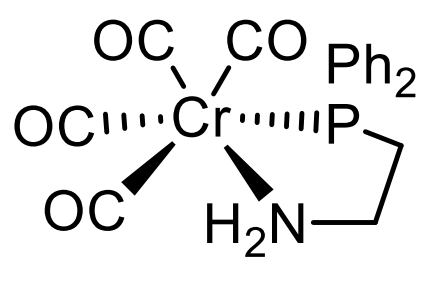

Cr-1

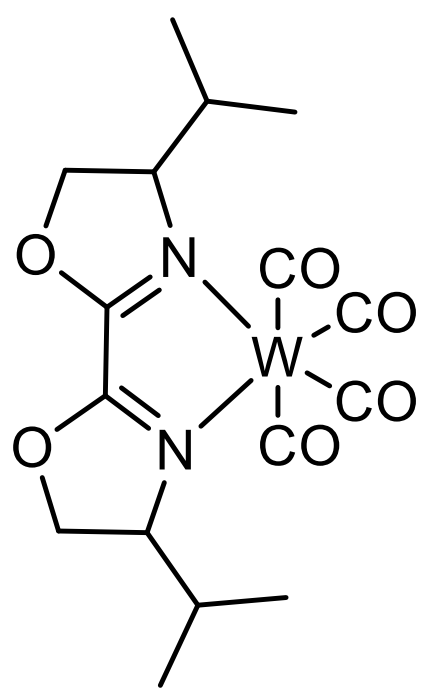

W-8

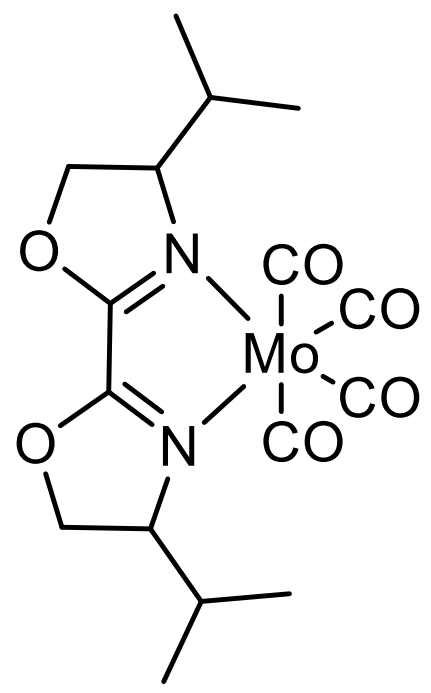

Mo-3

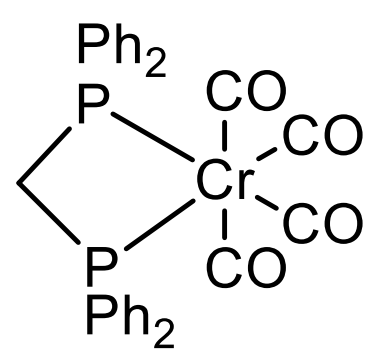

Cr-2

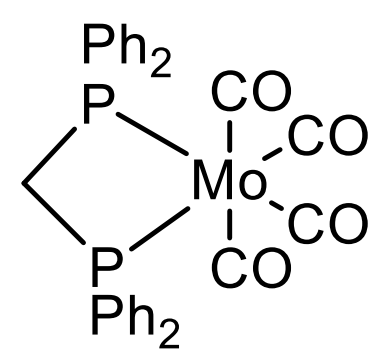

W-10

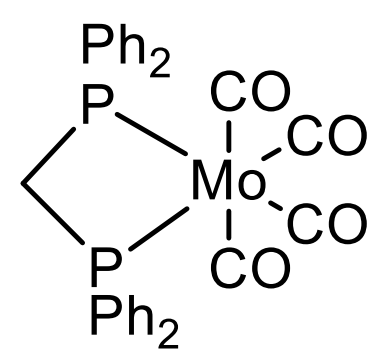

Mo-5

Ph = 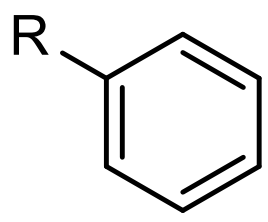

ACN = N≡C–CH₃

Figure S1: Structures of the herein investigated complexes.

S1.2 Chromatographic separation

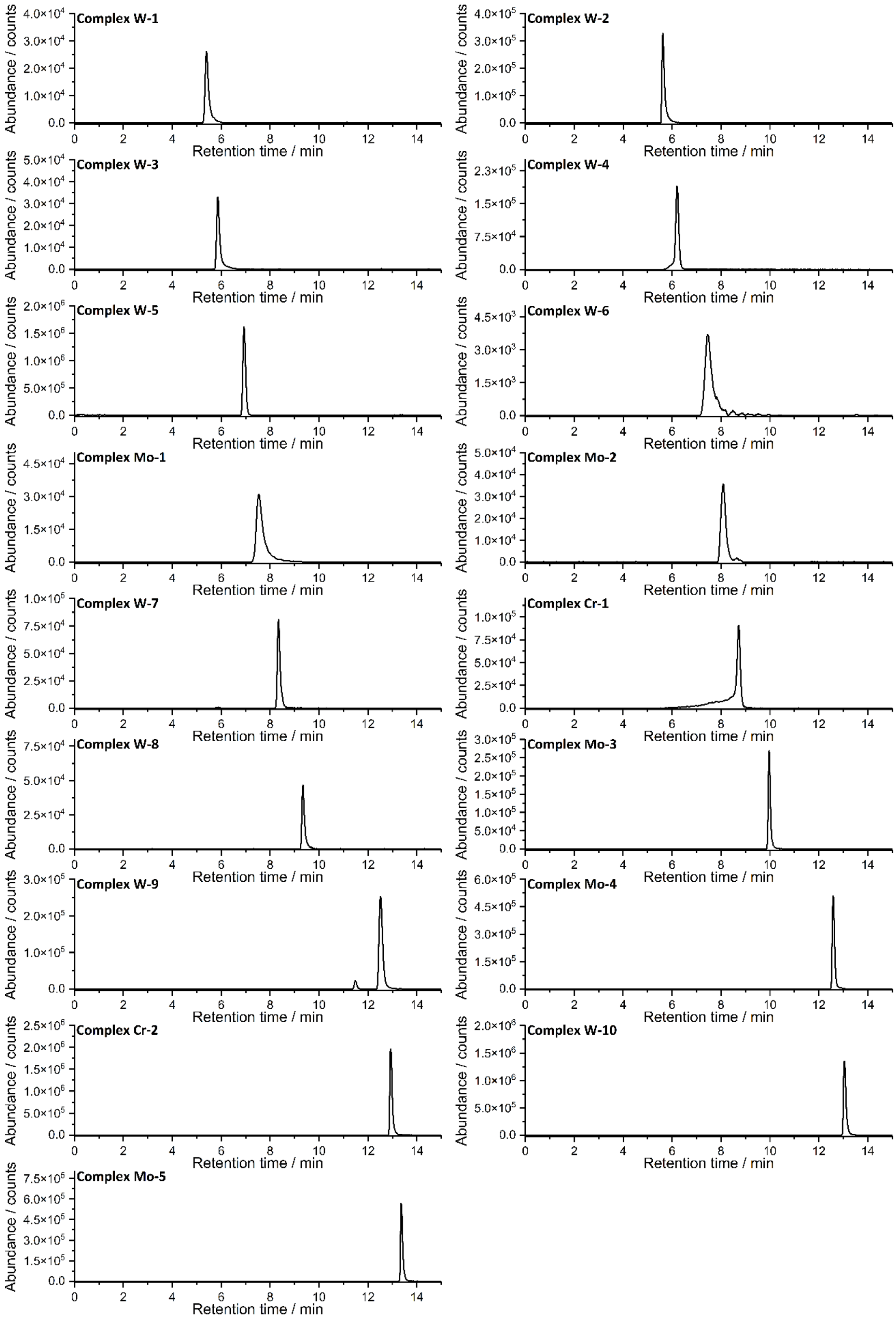

Figure S2: Extracted ion chromatograms of the herein investigated complexes.

Table S2: Charged species, drift times and $^{DT}CCS_{N2}$ of the herein investigated complex species (*n*=5).

Complex	Charged species	DT / ms	$^{DT}CCS_{N2}$ / Å^2	STDEV / Å^2
**W-1**	$[M]^+$	25.0	184.6	0.1
**W-2**	$[M]^+$	30.0	219.5	0.3
**W-3**	$[M-CO]^+$	32.8	240.0	0.3
**W-4**	$[M-ACN+H]^+$	20.5	153.2	0.2
**W-5**	$[M+H]^+$	22.0	163.3	0.2
**W-6**	$[M]^+$	36.9	269.9	0.1
**Mo-1**	$[M]^+$	36.2	264.9	0.2
**Mo-2**	$[M-CO+H]^+$	21.8	164.2	0.2
**W-7**	$[M]^+$	24.5	181.4	0.1
**Cr-1**	$[M]^+$	24.3	180.2	0.1
**W-8**	$[M+H]^+$	24.6	180.9	0.2
**Mo-3**	$[M-CO+H]^+$	23.5	173.7	0.3
**W-9**	$[M+H]^+$	28.2	206.0	0.1
**Mo-4**	$[M+NH_4]^+$	28.7	211.1	0.1
**Cr-2**	$[M+H]^+$	29.1	214.5	0.2
**W-10**	$[M+H]^+$	29.5	216.2	0.2
**Mo-5**	$[M+H]^+$	29.4	216.1	0.2

DT: drift time; $^{DT}CCS_{N2}$: collision cross section; STDEV: standard deviation; ACN: acetonitrile

S1.3 Isotopic pattern

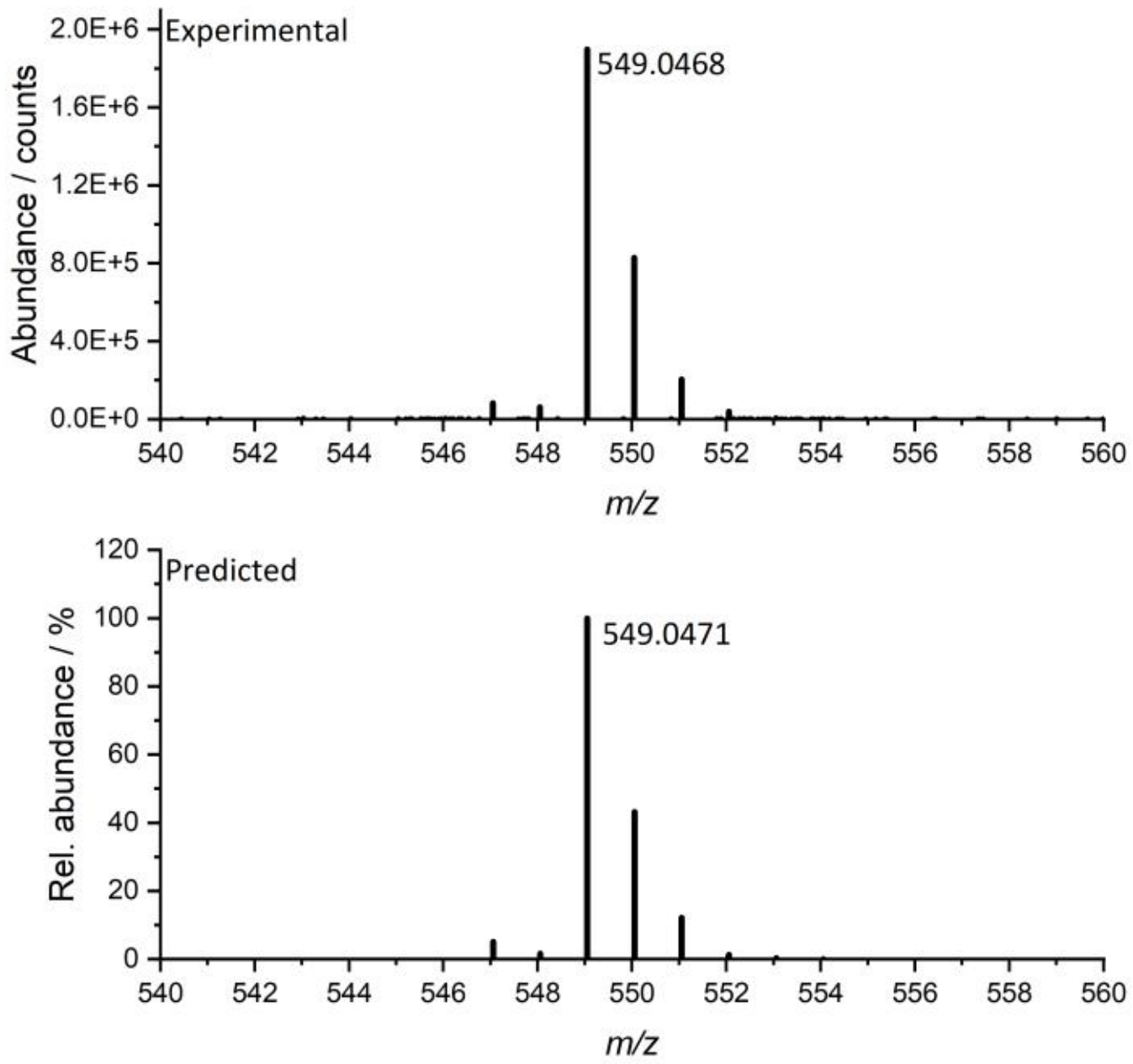

Figure S3: Experimental and predicted isotopic pattern for **Cr-2**. The isotopic pattern was calculated using *enviPat* [1]. Monoisotopic masses are indicated yielding an error of -0.6 ppm.

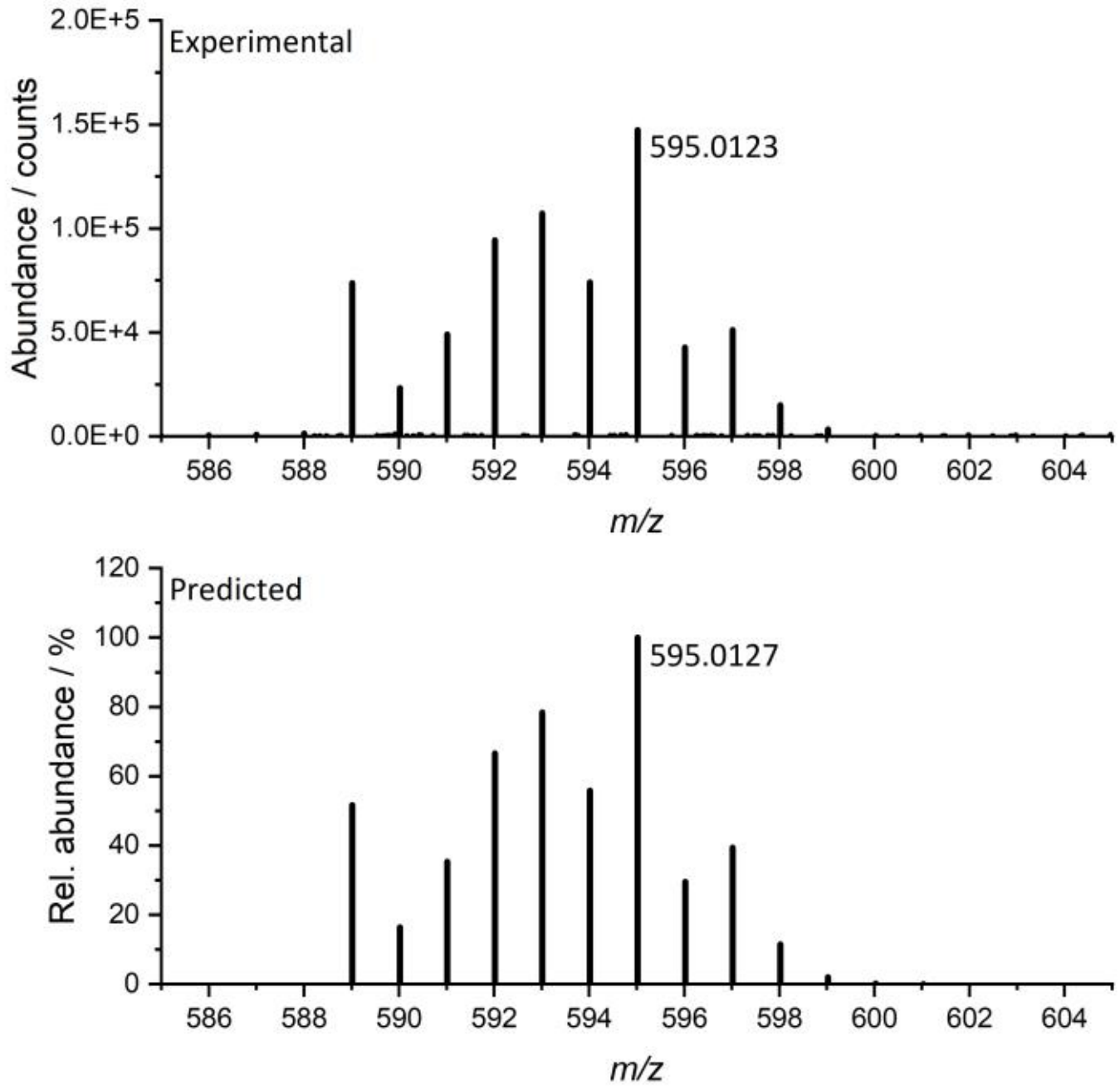

Figure S4: Experimental and predicted isotopic pattern for **Mo-5**. The isotopic pattern was calculated using *enviPat* [1]. Monoisotopic masses are indicated yielding an error of -0.7 ppm.

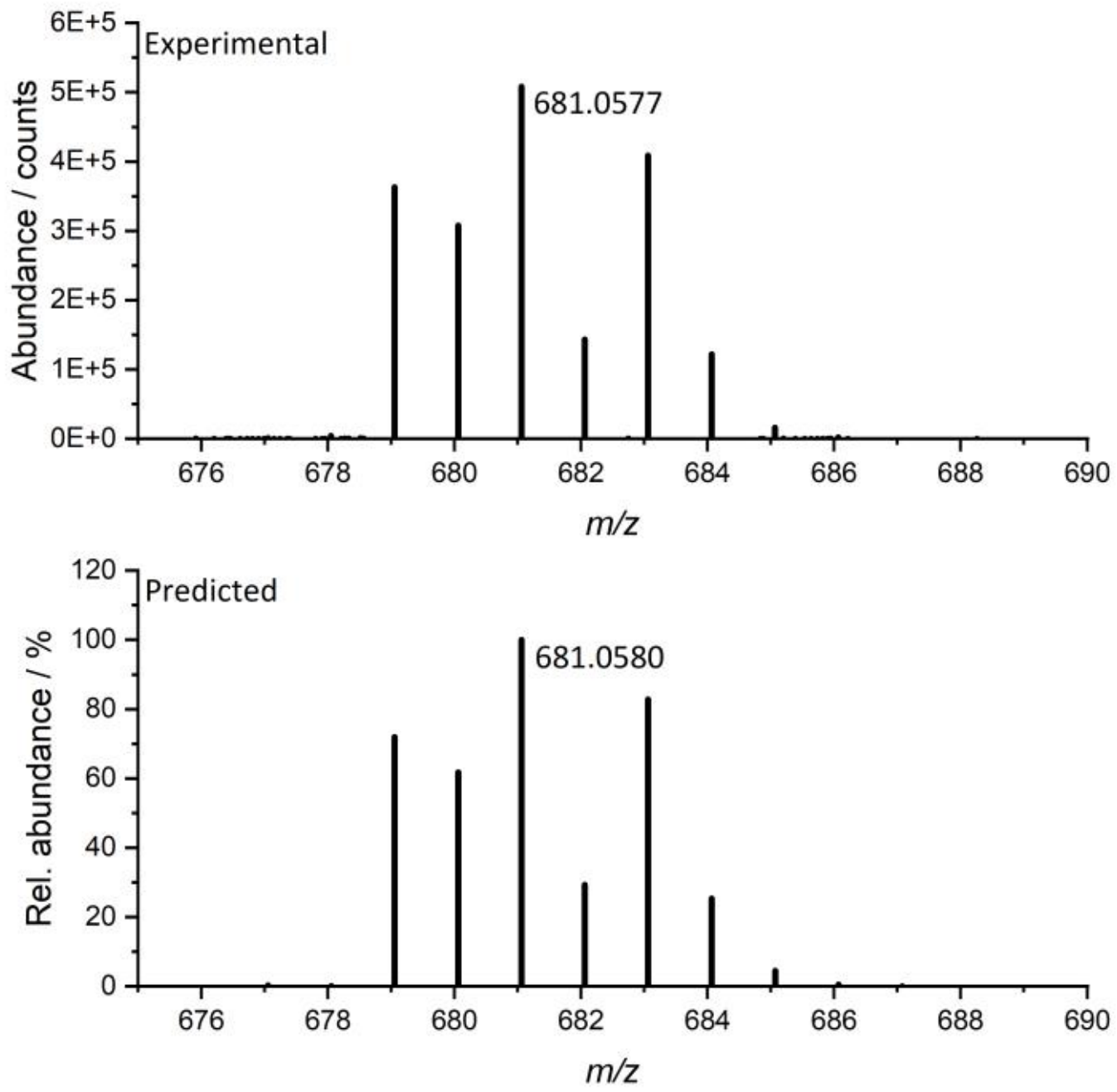

Figure S5: Experimental and predicted isotopic pattern for **W-10**. The isotopic pattern was calculated using *enviPat* [1]. Monoisotopic masses are indicated yielding an error of -0.4 ppm.

S2 Ion mobility measurements

S2.1 Trend of metal complexes in IM-MS

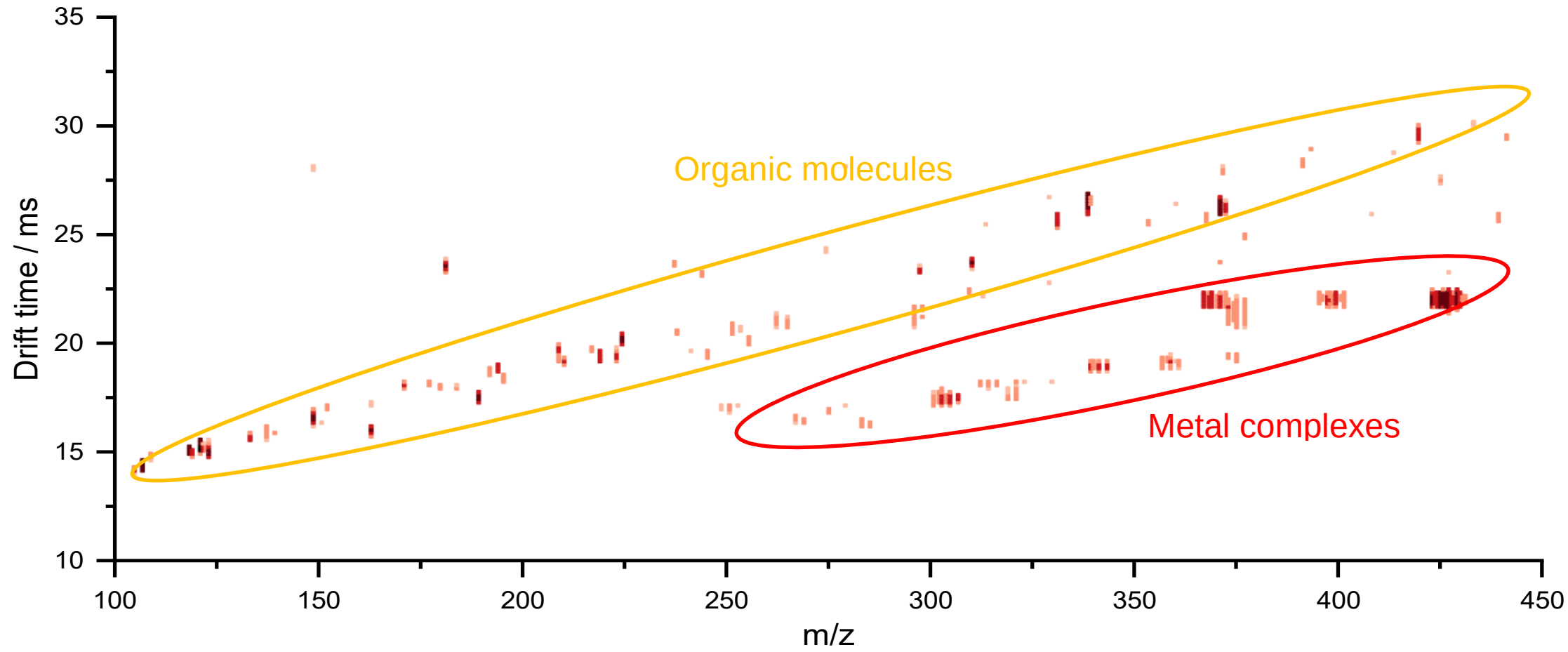

Figure S6: Drift time trends of organic molecules versus metal complexes. Metal complex signals deviate from the main organic molecule trend, appearing at shorter drift times for comparable *m/z* values.

S2.2 IM-MS data of unknown complexes

Table S3: Isotopologue pattern and assigned metal center for unknown complexes. Assignment according to the trends seen in Figure 7 for unknown products / by-products / contaminations of several W or Mo reaction mixtures.

Reaction mixture	*m/z*	RT / min	DT / ms	$^{DT}CCS_{N2}$ / Å²	Isotopologue pattern	Metal according to trend
**1 - Mo**	328.00	4.7	21.0	156.6	Mo	Mo
	540.07	5.9	27.1	199.1	Mo	Mo
	747.06	9.1	31.6	243.9	Mo	Mo
	696.13	7.8	32.0	234.2	Mo	Mo
	668.13	7.8	31.2	228.7	Mo	Mo
	640.14	7.8	31.0	226.8	Mo	Mo
	457.02	7.8	26.1	192.6	Mo	Mo
	547.08	7.8	28.4	208.4	Mo	Mo
**2 - Mo**	431.00	7.8	24.0	177.3	Mo	Mo
**3 - Mo**	386.89	5.8	20.5	152.3	inconclusive	W
	425.05	12.0	24.4	180.3	Mo	Mo
	481.03	12.0	25.7	189.8	Mo	Mo
**4 - Mo**	345.91	7.6	19.1	142.2	inconclusive	W
**5 - Mo**	321.92	4.8	19.9	148.3	inconclusive	Mo
**6 - W**	782.17	8.1	32.1	234.4	W	W
	663.13	7.4	28.8	210.9	W	W
	577.14	7.4	26.3	193.2	W	W
	489.08	7.2	23.2	170.7	W	W
	523.10	5.8	24.4	179.4	W	W
	412.03	5.8	20.6	152.3	W	W
	437.05	6.9	24.1	178.3	Mo	Mo

RT: retention time; DT: drift time; $^{DT}CCS_{N2}$: collision cross section;

S3 Complex degradation products

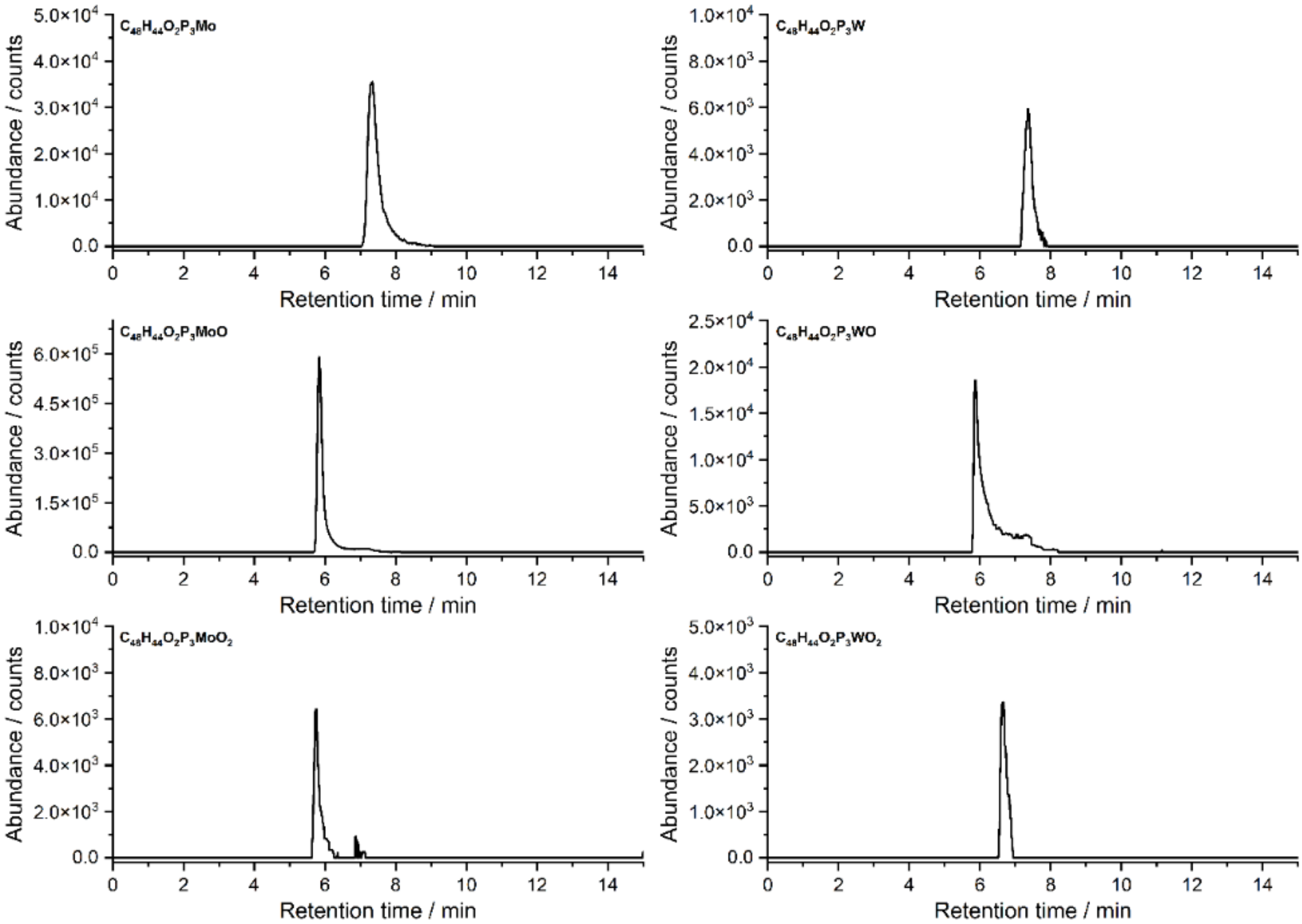

Figure S7: Extracted ion chromatograms of oxidized byproducts. Once and twice oxidized species of **Mo-1** (left) and **W-6** (right).

S4 References

1. Loos, M., Gerber, C., Corona, F., Hollender, J. & Singer, H. Accelerated Isotope Fine Structure Calculation Using Pruned Transition Trees. *Anal. Chem.* **87**, 5738–5744 (2015).
